# Effect of Gelation Temperature on the Molecular Structure and Physicochemical Properties of the Curdlan Matrix: Spectroscopic and Microscopic Analyses

**DOI:** 10.3390/ijms21176154

**Published:** 2020-08-26

**Authors:** Barbara Gieroba, Anna Sroka-Bartnicka, Paulina Kazimierczak, Grzegorz Kalisz, Izabela S. Pieta, Robert Nowakowski, Marcin Pisarek, Agata Przekora

**Affiliations:** 1 Department of Biopharmacy, Medical University of Lublin, Chodzki 4a, 20-093 Lublin, Poland; barbaragieroba@umlub.pl (B.G.); grkalisz@gmail.com (G.K.); 2 Department of Genetics and Microbiology, Institute of Microbiology and Biotechnology, Maria Curie-Sklodowska University, Akademicka 19, 20-033 Lublin, Poland; 3 Department of Biochemistry and Biotechnology, Medical University of Lublin, Chodzki 1, 20-093 Lublin, Poland; paulina.kazimierczak@umlub.pl; 4 Institute of Physical Chemistry, Polish Academy of Sciences, Kasprzaka 44/52, 01-224 Warsaw, Poland; ipieta@ichf.edu.pl (I.S.P.); rnowakowski@ichf.edu.pl (R.N.); mpisarek@ichf.edu.pl (M.P.)

**Keywords:** biopolymers, 1,3-β-d-glucan, vibrational spectroscopy, X-ray photoelectron spectroscopy, AFM microscopy, biomaterials

## Abstract

In order to determine the effect of different gelation temperatures (80 °C and 90 °C) on the structural arrangements in 1,3-β-d-glucan (curdlan) matrices, spectroscopic and microscopic approaches were chosen. Attenuated total reflection Fourier transform infrared spectroscopy (ATR FT-IR) and Raman spectroscopy are well-established techniques that enable the identification of functional groups in organic molecules based on their vibration modes. X-ray photoelectron spectroscopy (XPS) is a quantitative analytical method utilized in the surface study, which provided information about the elemental and chemical composition with high surface sensitivity. Contact angle goniometer was applied to evaluate surface wettability and surface free energy of the matrices. In turn, the surface topography characterization was obtained with the use of atomic force microscopy (AFM) and scanning electron microscopy (SEM). Described techniques may facilitate the optimization, modification, and design of manufacturing processes (such as the temperature of gelation in the case of the studied 1,3-β-d-glucan) of the organic polysaccharide matrices so as to obtain biomaterials with desired characteristics and wide range of biomedical applications, e.g., entrapment of drugs or production of biomaterials for tissue regeneration. This study shows that the 1,3-β-d-glucan polymer sample gelled at 80 °C has a distinctly different structure than the matrix gelled at 90 °C.

## 1. Introduction

**Figure 1:**
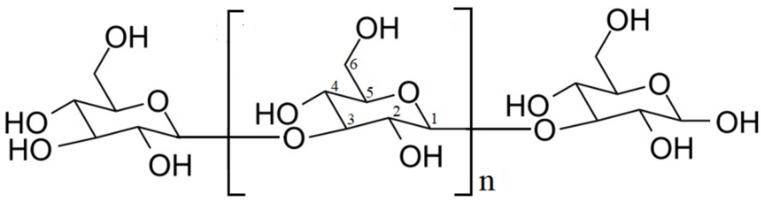
Chemical structure of 1,3-β-d-glucan (curdlan) [3].

Natural polymers are an extremely diverse class of molecules of high complexity consisting of repeating units. The most important and most numerous in nature of them are proteins, poly(nucleic acids) and polysaccharides characterized by a huge variety [1]. Glucans are a group of polysaccharides consisting of d-glucose monomers linked by glycosidic bonds [2]. They are found both in prokaryotes and eukaryotes and comprise the linear or side-chain branched (1→3, 1→2) and the (1→3, 1→6)-β-glucans [2,3]. Among them, curdlan has become of special interest, mainly due to its unique gel forming properties. It is a water-insoluble microbial exopolysaccharide produced by Alcaligenes faecalis var. myxogenes 10C3 strain, discovered by Harada et al. in 1966 during study of succinoglucan formation [4]. Curdlan has a simple, essentially linear homopolymeric structure. It is composed of d-glucose units with β-(1→3)-glycosidic linkage, but also may have a few intra- or inter-chain (1→6)-linkages (Figure 1) [5].

By changing the temperature and/or time of heating and the concentration of curdlan, gels with various strengths can be formed. Curdlan may create a thermoreversible, low-set gel when heated to 55 °C in aqueous solution or diluted alkaline solutions with further cooling or a stable thermoirreversible, high-set gel when heated to temperatures more than 80 °C in aqueous suspension [2,6]. These gelation properties have been associated with helical structure transformation of curdlan at different temperatures [7]. When heating, the triple-helical strands unwind to create single chains that can anneal to re-form a triple helical structure when cooling. At higher temperatures it forms more condensed rod-like triple helices [3]. Additionally, curdlan was shown by direct ultramicroscopic imaging to form macrocyclic and hairpin structures at low concentrations under DMSO treatment [8].

Due to its gelation ability at a 2–10 pH range, curdlan has found application in many fields, such as food, medicine and pharmaceutical industry [9]. It creates sterilizable, freezable and flavorless food gels, does not show toxicity and is approved by the Food and Drug Administration [10]. Curdlan can be used in novel, calorie-reduced products, because it is able to mimic the texture of foods containing fat [6,11]. Being a natural, biodegradable and biocompatible polymer, it is used in the engineering of biomaterials field for the production of 3D scaffolds or membranes for regenerative medicine applications [12]. β-glucan polymers and some of their aminated and sulphated derivatives are known as a biological response modifiers (BRMs) and have a variety of effects on the immune system by influencing macrophages, monocytes, neutrophils, dendritic cells and natural killer cells through interactions with soluble or cell surface receptors [2,13]. These effects include antitumor, antifungal, antiviral (anti-HIV), antibacterial and antiprotozoal activities. Curdlan derivatives may reduce hyperglycemia, hyperinsulinemia and hyperlipidemia, control diabetes, accelerate wound healing and possess anticoagulant, and antioxidant activities [13]. Therefore, they may be applied as a probiotic and vaccine adjuvant [14]. In the nanoscience field, it was demonstrated that the connection of curdlan gels and single-walled carbon nanotubes could supply innovative supernanostructures [15].

One of the most promising applications of 1,3-β-d-glucans is controlled drug delivery and entrapment [16]. Photopolymerized curdlan hydrogels have been used as drug delivery vehicles and gel encapsulation systems for bioactive macromolecules, which can control and prolong the release of drugs [17] and act as an enzyme immobilization matrix [18]. Drugs including indomethacin, salbutamol sulphate, prednisolone, and theophylline have been encapsulated in curdlan gels [16,19]. Moreover, curdlan suppositories are more often used in rectal administration by avoiding first pass clearance in the liver, unaffected by hypotonicity or isotonicity [20]. Another strategy is the encapsulation of drug loaded nanoparticles in glucan-based microspheres, which play a role as a specific target for uptake by macrophages and dendritic immune cells [21]. These microspheres can be used for delivering various particles, e.g., proteins such as model bovine serum albumin (BSA) [17], polynucleotides e.g., DNA, siRNA [22], and other small molecules [23]. Moreover, curdlan (as an immunotherapeutic adjuvant) and polyethylene glycol (PEG) have been applied as a model platform for doxorubicin, a chemotherapeutic drug [24]. Another application of curdlan includes preparation of grafted hydrogel polymer for delivery of all-trans retinoic acid (ATRA) to a hepatoma cell line (HepG2) by modification of the carboxymethylated curdlan (CM-Curdlan) with lactobionic acid on the backbone followed by its conjugation to sulfonylurea [25].

It is well known that many agents influence the chemical and mechanical properties of curdlan gels. They include concentration, dispersing method, temperature, time, and rate of heating [26]. Because curdlan seems to be a promising drug carrier, the main emphasis of this research focuses on the effect of gelation temperature on the properties of the polymer films, including hydrophilicity/hydrophobicity, cross-linking density, and molecular structure. The aim of this study was to determine the physicochemical and structural characteristics of 1,3-β-d-glucan polymer matrices gelled at two different temperatures (80 °C and 90 °C). These features were analyzed by the combination of Fourier transform infrared spectroscopy (ATR FT-IR), Raman spectroscopy, X-ray photoelectron spectroscopy (XPS), contact angle measurements, atomic force microscopy (AFM), and scanning electron microscopy (SEM).

## 2. Results

### 2.1. ATR FT-IR Spectroscopy

**Figure 2:**
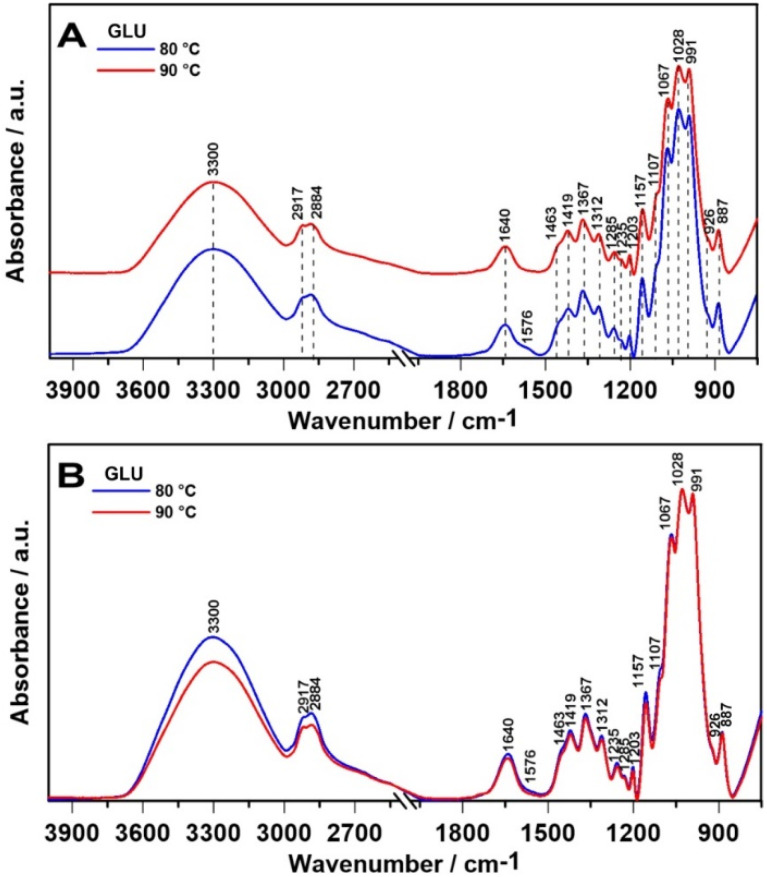
The relative intensity of ATR FT-IR (attenuated total reflection Fourier transform infrared spectroscopy) spectra of 1,3-β-d-glucan (**A**) and spectra normalized to the highest intensity band (1028 cm^−1^) arising from C-O vibration (**B**).

The ATR FT-IR spectrum of 1,3-β-d-glucan (Figure 2A,B) showed its characteristic band at 887 cm^−1^ which correlates with the presence of β-glycosidic bond, i.e., C-H deformation mode [7,12,27]. Absorption peaks at 3300 cm^−1^ indicate OH stretch, as the free hydroxyl groups absorb in the region of 3650–3500 cm^−1^ [27], while the peaks at 1419 and 1367 cm^−1^ arose from bending modes of CH_2_, CH, and OH [28]. The next bands at 2884 and 1203 cm^−1^ come from C-H stretching and CH_2_OH stretching of the glucose side chains, respectively [27]. Intense overlapped bands in the region of 800–1300 cm^−1^ correspond to C-O, C-C stretching, and COH bending modes [28]. The major bands appearing in the FT-IR spectra of 1,3-β-d-glucan and their assignment to particular groups are presented in the Table 1.

**Figure 3:**
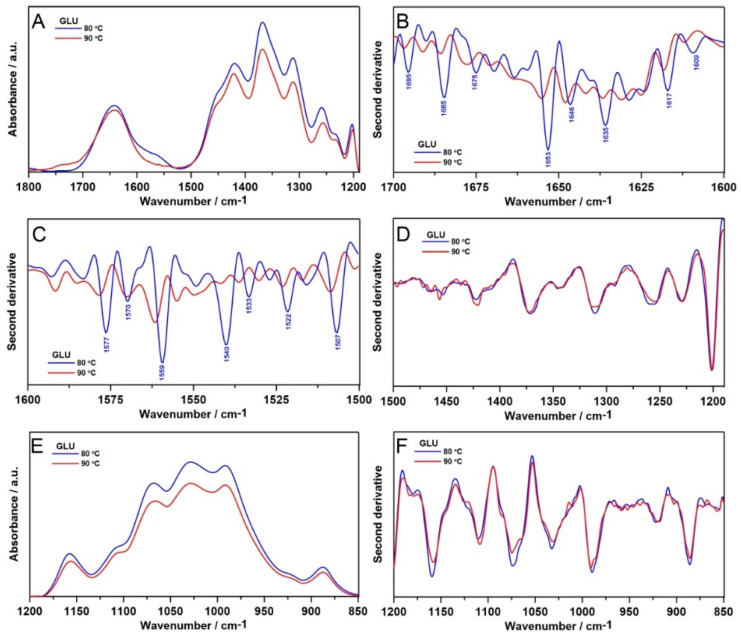
The relative intensity of ATR FT-IR spectra in the 1800–1200 cm^−1^ range (**A**), the second derivatives of the ATR FT-IR spectra in the range of 1700–1600 cm^−1^ (**B**), 1600–1500 cm^−1^ (**C**), 1500–1190 cm^−1^ (**D**, fingerprint region specific for carbonyl groups in samples), the relative intensity of ATR FT-IR spectra in the 1200–850 cm^−1^ range (**E**, specific region for ring-structured carbohydrates) and the second derivatives in this spectral range (**F**).

To analyze more in depth the structural changes in the 1800–1200 cm^−1^ and 1200–850 cm^−1^ regions, the relative intensities of ATR FT-IR spectra are shown in Figure 3A,E, respectively. Next, the second derivatives were determined in analyzed spectral regions (Figure 3B–D,F) in order to investigate the molecular modifications and secondary structure alterations of polymer chains in the tested samples.

The ATR FT-IR 1800–1200 cm^−1^ bandwidth shows a similar pattern for these samples, except the appearance of a new band at 1576 cm^−1^ in 80°C sample (Figure 3A), attributed to carboxylate ion [29]. A different molecular arrangement in this temperature is also visible in the second derivative course. Both 1700–1600 cm^−1^ (Figure 3B) and 1600–1500 cm^−1^ (Figure 3C) absorption profiles are very susceptible to variations within secondary structure of polymer chains [31]. In the 80 °C sample, additional bands appeared assigned to diverse secondary structures: 1695 (aggregates), 1685 (antiparallel β-sheets), 1675 (turns), 1653 (α-helices), 1646 (unordered), 1636 (parallel β-sheets), 1617 and 1609 cm^−1^ (aggregates) [32]. A similar situation is observed in the 1600–1500 cm^−1^ region: new bands were detected in the 80 °C sample: 1577, 1570, 1559 (aggregates and unordered), 1540 (α-helices), 1533 (parallel β-sheets), 1522 and 1507 cm^−1^ (antiparallel β-sheets) [33,34]. In turn, spectral range between 1500 and 1190 cm^−1^ is characteristic for a mixed CH_2_, CH_3_ and CO bending vibrations [31], but no considerable temperature-dependent changes were observed (Figure 3D). Spectra in the 1200–850 cm^−1^ region (connected with cyclic structures of carbohydrates [35]) are similar for all specimens, but matrix gelled at 80 °C is characterized by higher intensity of absorbance. Furthermore, no significant alterations in corresponding second derivative function (Figure 3F) have been found. These results indicate a totally different molecular organization of curdlan gelled at 80 °C in comparison with 90 °C samples.

### 2.2. Raman Spectroscopy

**Figure 4:**
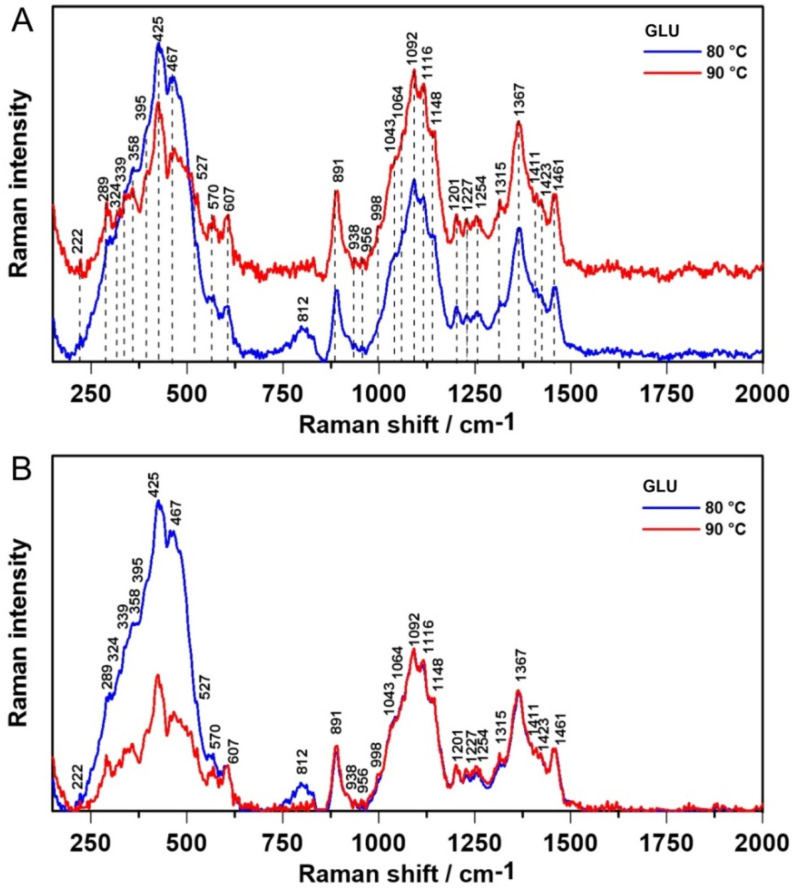
The relative intensity of Raman spectra of 1,3-β-d-glucan (**A**) and spectra normalized to the band at 1092 cm^−1^ (**B**).

Raman spectra of 1,3-β-d-glucan matrices cross-linked at temperatures 80 °C and 90 °C are shown in Figure 4A. For a more consistent comparative evaluation, the 1,3-β-d-glucan spectra were normalized to a band at 1092 cm^−1^ (Figure 4B) attributed to the stretching CC and CO stretching modes [36]. The band at approximately 1148 cm^−1^ is assigned to C-O-C vibrations of the glycosidic bond [28]. The band at 1367 cm^−1^ is connected with angular deformation of the CH and COH groups [7]. The previously mentioned band at 1092 and shoulder band at ~1148 cm^−1^ are characteristic for β-glucans. Intense highly overlapped Raman 990–1200 cm^−1^ bandwidth comes from COC and CC stretching vibrations of polysaccharides. The bands in the 1200–1440 cm^−1^ spectral range mainly arise from in-plane ring deformation modes and OH and CH bending vibrations. Eventually, the band at 1461 cm^−1^ is attributed to CH_2_ in-plane bending in CH_2_OH of the molecule [7]. Table 2 contains the most important bands recorded in 1,3-β-d-glucan spectra.

**Figure 5:**
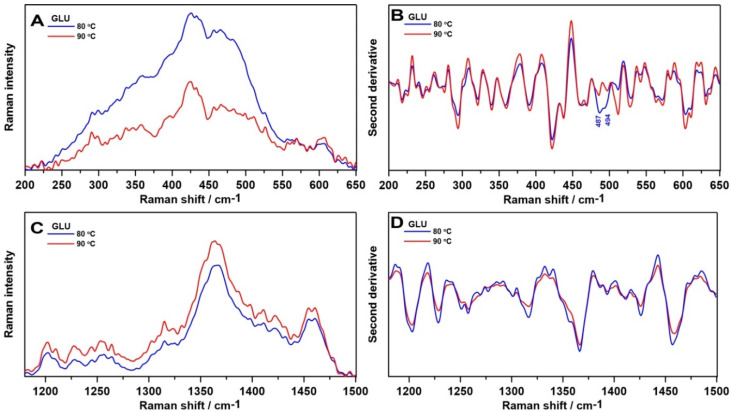
The relative intensity of Raman spectra in the 200–650 cm^−1^ (**A**) and 1180–1500 cm^−1^ range (**B**), and the second derivatives of the Raman spectra in these ranges (**C**,**D**, respectively).

A closer look at the 200–650 cm^−1^ and 1180–1500 cm^−1^ regions gives the relative intensity Raman spectra presented in Figure 5A,C, respectively. As in the ATR FT-IR spectra, more information can be obtained by determination of the second order derivative spectra in these spectral ranges (Figure 5B,D, respectively), which enable us to track the changes in the intensity variations and may resolve broad bands into individual components leading to increased accuracy.

The biggest differences between the samples were noticed in the 200–650 cm^−1^ region (Figure 5A), which contributed to skeletal deformational and bending frequencies [7]. The 1,3-β-d-glucan sample gelled at 80 °C had higher Raman intensity. Moreover, in the second derivative course, two new bands were revealed: 487 cm^−1^ attributed to a skeletal distortion (CH def.) and 494 cm^−1^ ascribed to ring vibration of carbohydrates [37]. The range between 1180 and 1500 cm^−1^ is assigned to the conformation of the glycosidic linkages (1200–1300 cm^−1^) and CCH, OCH, COH, and HCH skeletal stretching [38]. There were no notable changes in the recorded spectra (Figure 5C) and inconsiderable minor shifts in second derivative course (Figure 5D). These may suggest different skeletal conformations in samples gelled at 80 °C. These results remain in accordance with FT-IR spectroscopic analysis.

### 2.3. X-ray Photoelectron Analysis

**Figure 6:**
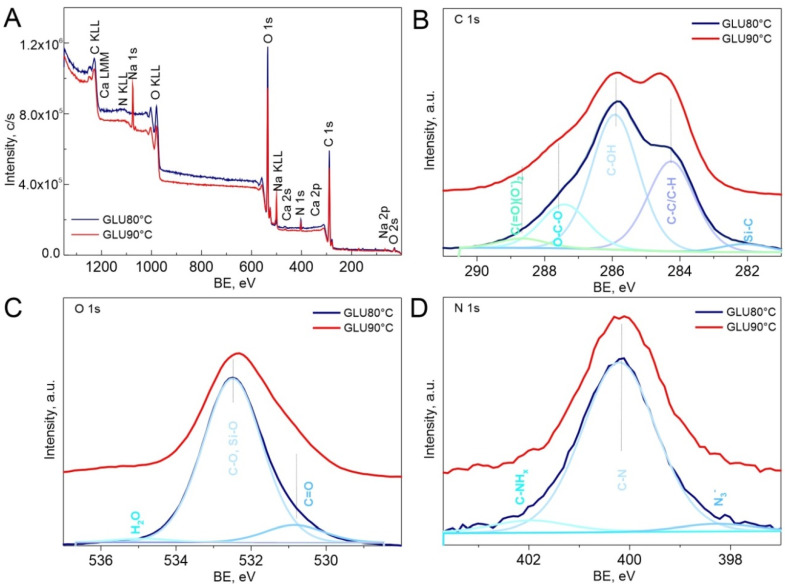
XPS spectra for glucan samples: (**A**) wide scan spectra and (**B**) C 1s, (**C**) O 1s, (**D**) N 1s core level spectra.

X-ray photoelectron spectroscopy was used to determine the chemical state of the surface elements in the studied samples. Figure 6 presents the full survey and core level spectra, respectively, of glucan samples gelled at 80 °C and 90 °C. In Figure 6A, in the wide scan spectra of the specimens the peaks at 285, 398, 533, 1070 and 345 eV for C, N, O, Na 1s and Ca 2s were detected [39].

The glucan samples consisted mainly of the elements C, N, and O, with smaller contribution of elements such as Na, Ca, S, P, Si. Table 3 presents the calculated chemical composition of polymeric samples.

The atomic ratio of C to O at the surface of glucan samples was around 2, which is above the ideal value of ca. 1.5 expected for glucan. The C to N ratios are equal 23 for GLU 80 °C and GLU 90 °C samples, indicating that nitrogen has been introduced into investigated surfaces of glucan samples during their synthesis and film preparation. Undoubtedly, the higher temperature treatment after sample preparation results in higher nitrogen content in the investigated film.

The C1s core level spectra deconvolution of the glucan sample treated at 90 °C shown in Figure 6 revealed five peaks at binding energies of 282.7, 284.6, 286.2, 287.8, and 289.0 eV, which were assigned to Si-C-O at 282.7 eV, hydrocarbon (C-H/C-C) at 284.6 ± 0.1 eV, alcohol/ether/amine (C-OX) at 286.2 ± 0.1 eV, acetal (O-C-O) at 287.8 ± 0.1 eV and O=C-OH group at 289.1 ± 0.1 eV [40]. The spectra look quite different for the sample GLU 80 °C. For this sample, spectra showed an increase in the amount of alcohol/ether/amine (C-OX) surface species, with a peak observed at 286.3 eV, confirming the strong wrapping of carbon bonds. The acetal (O-C-O) functionality detected at 287.8 eV is also of higher intensity comparing to the GLU 90 °C sample, which most likely is indicative to some extent of surface functionality changes for the intermediate temperature. This correlates well with AFM study, showing that surface chemical state and functionality changes correspond to morphology and topography sample changes evoked by temperature [41].

The N1s region of the XPS spectrum of glucan samples is shown in Figure 6D. The results indicated the peaks at 398.1 ± 0.2, 399.9 ± 0.2 and 402.3 ± 0.2 eV correspond to the assignments of C-N, -NH- and -NH_x_ groups, respectively. The nitrogen content stayed stable for both GLU 80 °C and GLU 90 °C samples, thus suggesting that the degree of deacetylation during temperature increase is minimal, if any. Analyzing the C-N/C-NH_x_ peak area ratio of GLU 80 °C and GLU 90 °C indicates that temperature treatment results in nitrogen-enriched polysaccharides, with more C-N detected on the surface [42].

The O1s region contains peaks assigned to C=O (531.3 eV), C-O (532.8 eV), and H_2_O adsorbed on the GLU samples surface (535. 8 eV).

The shapes of the N1s and O1s peaks in the spectra of both samples remain similar, suggesting that the identities and distributions of the most abundant surface species in these materials are similar and depend most likely on the surface structure and topography, which may favor the formation of certain species/surface functionalities to balance the system’s surface charge. These data agree well with the spectroscopic investigations, which indicate the glucan matrices caused modifications in the molecular arrangement.

XPS revealed the changes in molecular composition of prepared polymers. In the glucan sample treated at 80 °C, N was detected in the highest amount while Ca, Na, P and S surface concentrations were negligible.

### 2.4. Contact Angle Measurements

**Figure 7:**
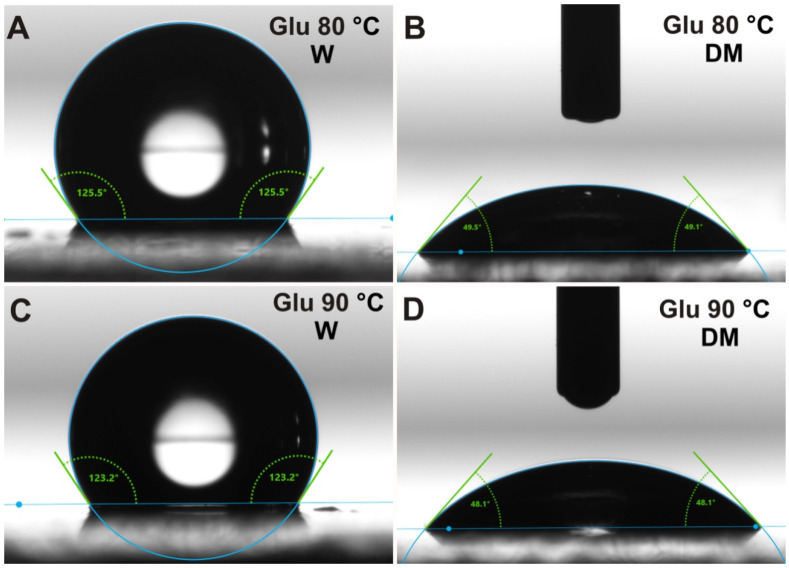
Images presenting water (W) (**A**,**C**) and diiodomethane (DM) (**B**,**D**) contact angle measurements for glucan matrices gelled at 80 °C (**A**,**B**) and 90 °C (**C**,**D**).

**Figure 8:**
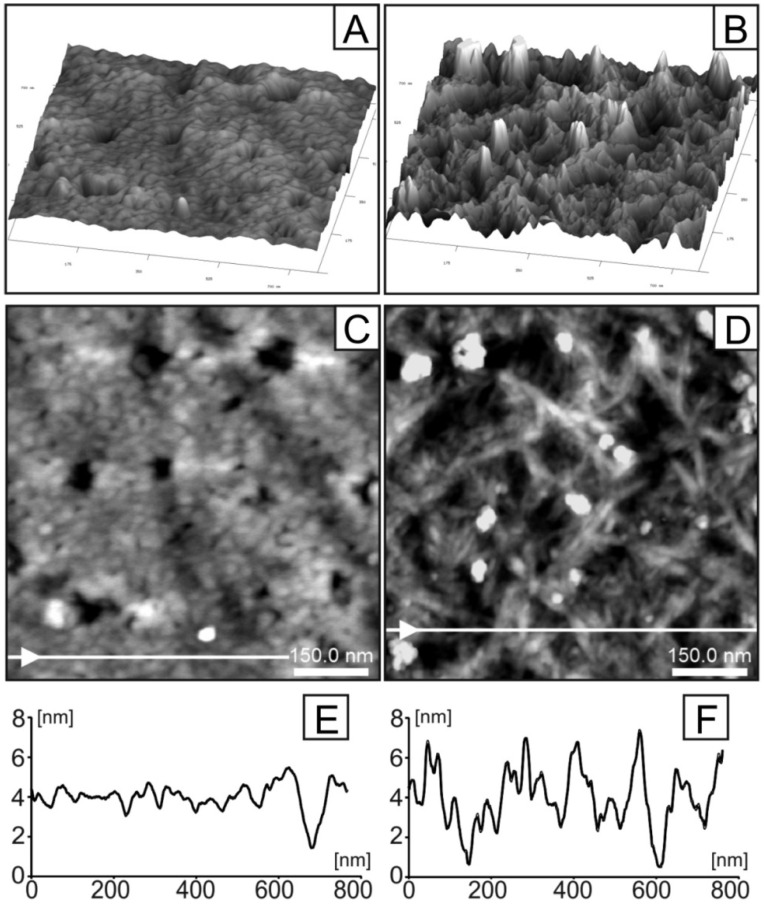
AFM (atomic force microscopy) images (**A**–**D**) and cross-sectional profiles measured along the white lines shown in Panels C and D (**E**,**F**) of GLU layer prepared at different temperatures: 80 °C (**A**,**C**,**E**) and 90 °C (**B**,**D**,**F**). Scanning area: (**A**–**D**) 780 × 780 nm^2^. Arrows on the white lines shown in Panels C and D define scan direction.

**Figure 9:**
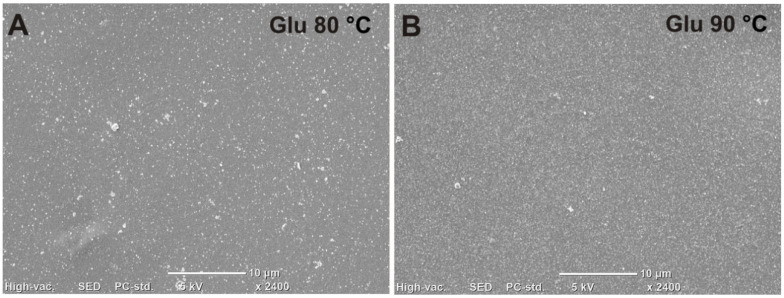
SEM (scanning electron microscopy) micrographs presenting surface morphology of glucan matrix gelled at 80 °C (**A**) and 90 °C (**B**).

Images presenting water and diiodomethane contact angle measurements may be seen in Figure 7. Water contact angles for both tested glucan matrices were high (indicating hydrophobic character) and similar: 125.01° ± 2.84° and 123.78° ± 2.61° for samples gelled at 80 °C and 90 °C, respectively. Diiodomethane contact angles were also comparable: 48.93° ± 1.72° and 48.67° ± 1.80° for samples gelled at 80 °C and 90 °C, respectively. This is a normal phenomenon since both matrices have the same chemical composition. However, the glucan gelled at 90 °C showed a slightly lower water contact angle (higher wettability) compared to the sample gelled at 80 °C. It is well known that not only surface chemistry but also topography has a great impact on the wettability of biomaterials [43,44]. Biomaterials revealing more rough surfaces generally show a higher wettability compared to more smooth surfaces. Therefore, the observed slight differences in the wettability between the matrices gelled at different temperatures most likely resulted from their altered topography. Our assumption found the reflection in AFM and SEM visualization that showed higher roughness of glucan matrix gelled at 90 °C compared to the sample heated at 80 °C (Figure 8A,B and Figure 9A,B). Interestingly, despite the hydrogel character of the matrices, they were found to be hydrophobic due to a large share of dispersive component, as was demonstrated by the surface free energy calculations (Table 4). Nevertheless, it is a well-known and normal phenomenon that during drying of the hydrogel sample, the polar groups are hidden inside the material, significantly decreasing the polar component of the surface free energy which results in decreased wettability [30].

### 2.5. Surface Topography Imaging

Figure 8 presents representative AFM images of GLU layers prepared at 80 and 90 °C (images: A, B (3D presentation) and: C, D (2D), respectively). Moreover, the presentation is supported by profiles of selected lines (E, F) marked by white arrows and lines in images (C, D). Comparison of microscopic observations evidently shows the important role of the preparation temperature on topological features of the investigated material. The surface of the samples prepared at the higher temperature (90 °C) are characterized, as expected, by a fibrous structure. In these cases, single fibers are easily distinguished on AFM images as bright lines. The width of the observed longitudinal structures are in the range 12–18 nm (however this dimension can be overestimated by the shape of the AFM tip). The fibers are randomly distributed and mutually intertwined. As a consequence the surfaces of these samples are relatively rough. They are additionally decorated by randomly distributed grains of sizes between 50–70 nm and high 5–10 nm (visible in the images as bright spots). The surface of the sample prepared at temperature 80 °C is, however, significantly different. The surface of this sample is much more smooth in comparison to the sample prepared at 90 °C (compare 3D images (Figure 8A,B) and line profiles (Figure 8E,F). This observation indicates that the fibers in the layer prepared at 80 °C are significantly more densely packed. The characteristic features of this surface are randomly distributed pores of the size in the range 30–70 nm (visible in image Figure 8C as dark spots).

Surface morphology of the glucan matrices gelled at different temperatures was also visualized by SEM. The performed observation confirmed higher roughness of the glucan matrix gelled at 90 °C compared to the sample heated at 80 °C (Figure 9). Surface morphology of the sample gelled at 90 °C was characterized by noticeably greater granulation compared to the sample gelled at lower temperature.

## 3. Discussion

Fourier transform infrared (FT-IR) and Raman spectroscopies are efficient techniques in providing direct and detailed data about the molecular and chemical composition of biological specimens [45,46,47]. They are very helpful in understanding and explaining biological processes, especially at the molecular level [48]. These vibrational spectroscopies are also commonly used as complementary techniques for the structural analysis of various polysaccharides [49], so they will also be immensely useful in curdlan research. Some overlaid or weak bands that cannot be recorded by FT-IR spectroscopy can be detected by applying Raman spectroscopy and inversely [7]. In the case of glucans, FT-IR spectroscopy is especially susceptible to the anomeric configuration and position of glycosidic linkages and Raman spectroscopy is particularly advantageous in detecting symmetric, non-polar groups, such as C-C and C=C in curdlan structure [28]. Hence, the combination of these complementary techniques, as done in this study, enables a more comprehensive approach for analyzing polymer molecular arrangement and provides more profound chemical information about functional groups and their vibrational modes.

In turn, X-ray photoelectron spectroscopy is one of the most powerful and widely used surface-sensitive quantitative spectroscopic techniques to determine the compositions and chemical species in their near-surface regions [40]. Surface features, which include among others chemical composition, topography, surface energy, and wettability, are especially crucial in biopolymer characterization because they directly influence cell adhesion and proliferation (thereby matrices biocompatibility) and may limit their biomedical applications [50]. The additional insight into the structure–function relationships of biopolymer samples is provided by atomic force microscopy, which is undoubtedly the most versatile and potent microscopy technique for analyzing samples at nanoscale. AFM visualizes the three-dimensional topography of a polymer sample surface by scanning the cantilever over a region of interest, in real time, under physiological conditions, and with minimal sample preparation. Measurements obtained by AFM provide the physical properties of the specimen, such as molecular interactions, surface hydrophobicity and charges, and also mechanical characteristics [51]. Tests on a contact angle goniometer were conducted to evaluate whether observed changes between the matrices, with respect to their chemical properties and topography, affected their wettability and surface free energies. The combination of these techniques allowed us to obtain full and comprehensive knowledge on the 1,3-β-d-glucan samples surface.

Natural polymers, like polysaccharides, that exist abundantly in nature, have recently gained a lot of interest due to the increasing problem of ecological degradation and wide spectrum of economic use, i.e., in the food, packaging, and medical industries. They are also economically advantageous in comparison with their synthetic non-renewable counterparts [52].

Based on the literature, 1,3-β-d-glucan seems to be particularly promising candidate for applications in the engineering of biomaterials as drug delivery systems or scaffolds for cell growth [16] and in order to fulfil its role, it must possess appropriate properties. The cross-linking density of curdlan matrices is a prevalent factor determining the drug release rates and mechanism [53]. Therefore, more densely cross-linked curdlan films may find application as a vehicle for sustained release of the drugs over a prolonged period of time, while being more resistant to enzymatic degradation [54,55]. In turn, curdlan matrices with lower cross-linking densities can be applied in the treatment of acute invasive infections, causing a burst release of a large dose of the drug. An example is curdlan-containing dressings that can be used to treat infected diabetic wounds [56]. Our research has shown that the sample cross-linked at 80 °C was characterized by denser cross-linking than the sample gelled at 90 °C, thereby indicating different adhibition in the biomedical and pharmaceutical fields.

We have observed a significantly different molecular structure in the matrix gelled at 80 °C confirmed by all applied biophysical techniques in this study. The sample cross-linked in this temperature is characterized by a different secondary structure. Furthermore, changes in the skeletal conformation (strong wrapping of carbon bonds, appearance of additional CN group) and distortion in aromatic rings have been detected. Moreover, the 80 °C specimen turned out to have more densely packed fibers with randomly distributed smaller sized pores (30–70 nm vs. 50–70 nm) in comparison with the matrix gelled at 90 °C. This indicates a smoother surface of the 80 °C sample. The clearest difference concerns the secondary structure of polymer chains. Second-order derivatives of FT-IR spectra showed that in the sample cross-linked at 80 °C, bands appeared attributed to α-helices (1540 and 1653 cm^−1^), which were absent in sample gelled at 90 °C. According to the literature, the conformation of dense cross-linked curdlan gel may consist of mere single- or triple-helical structures or a combination of single- and triple-helical structures with different water-binding capacity. These dissimilarities were presumably attributed to the different patterns of hydrogen bonding along with conceivable hydrophobic interactions associated with variations in chain mobility [3]. Helical forming polysaccharides are prone to aggregate, creating a structure of greater complexity and denser package [57] (aggregates were detected in the second derivative FT-IR spectra of 80 °C specimen at 1577, 1570, 1559 and 1609, 1617 cm^−1^). A more dense package of the sample gelled at 80 °C was also confirmed by XPS analysis, where strong wrapping of carbon bonds was found and visualized by AFM and SEM images. Moreover, lower roughness of the GLU 80 °C glucan film was demonstrated on 3D AFM data (Figure 8A) and the corresponding profile image (Figure 8E). As shown in Figure 8E,F, comparing GLU 80 °C vs. GLU 90 °C, the film roughness differed by 400% comparing both curdlan films. Considering identical GLU synthesis protocols, the differences observed in physicochemical structures must have originated from the structural differences and single moieties package/cross-linking and/or were most likely a combination of single- and triple-helical structures with different water-binding capacities. Figure 8 shows the differences not only in topography but also in material porosity, with spherical-like pores in GLU 80 °C vs. complex porous structure in GLU 90 °C. This may be indicative of the structural density and cross-linking differences in obtained films. The more complex porosity could be indicative of a less compact structure with enhanced hydrophilic properties. During film preparation and drying, dehydration would be more easily attained for less dense material, which was confirmed by XPS measurement, where the ratio of C-OH: (C-C+C-H) bond decreased with gelation temperature (Figure 6). It is, however, worth mentioning that during drying of the hydrogel sample, the polar groups were hidden inside the material, significantly decreasing the polar component of the surface free energy which results in decreased wettability, which is also shown in Figure 7 by contact angle measurements for glucan matrices.

The temperature of curdlan gelation influences many parameters, such as gel strength, fibrillar structure, and its reversibility. In the initial stages of heating process, the breakage of hydrogen bonds is required for gel formation and after cooling, the firm gel is formed due to the formation of hydrogen bonds. Mamaril et al. found that at temperatures <60 °C the gel formed by 1,3-β-d-glucan was too soft to determine its strength with the use of the curdmeter, but with increasing temperature the gel strength was also higher, and between 60° and 80 °C it was gradual, achieving the highest strength at >90 °C [58]. It was demonstrated that depending on the heating temperature, curdlan can create two types of gels: a high-set thermal non-reversible gel (~80 °C) and a low-set thermal reversible gel (~55 °C) [3]. This gelation ability has been connected with curdlan’s helical structure transformation from a compound of single helices and loose intertwined triple helices at room temperature to more condensed rod-like triple helices at higher temperatures. Curdlan low-set gels obtained by heating (~60 °C) showed rod-like fibrillar structures and the microfibrils were wider for curdlan gels formed at 90 °C and the widest at 120 °C, which is attributed to decreased crystallinity [59]. We examined two temperatures of gelation: 80 and 90 °C, and the lower studied temperature (80 °C) results in unique properties of the curdlan polymer sample. Taking into account non-reversibility, dense package of gel, less porosity, and strong chemical interactions within the molecular structure at this temperature of gelation, this form of curdlan matrix seems to be the most stable and resistant to external factors, which can be of great importance in biomedical industry. To sum up, our data demonstrate that a spectroscopic approach coupled with microscopic visualization can be successfully applied to determine the molecular structure and surface physicochemical properties of polysaccharide polymer matrices. The analytical techniques we have employed for biochemical analysis may find application in various biomedical areas for designing polymer-based biomaterials for regenerative medicine, tissue engineering, controlled drug delivery and release, and (bio)nanotechnology. Thus, this opens the possibility of using non-invasive spectroscopic and optical methods to optimize the manufacturing process of biomaterials and monitor matrices properties during production in different conditions, i.e., temperature, pH, concentration, and use of organic solvents. Nevertheless, in order to determine the molecular organization of curdlan matrices more precisely, additional methods should be used, such as NMR (nuclear magnetic resonance) spectroscopy, thermal analysis (DSC, differential scanning calorimetry and thermogravimetry), mass spectrometry, X-ray diffraction, and cryo-electron-microscopy.

## 4. Materials and Methods

### 4.1. Preparation of 1,3-β-d-glucan Samples

The thin polysaccharide films were produced by spreading of 8% (*w*/*v*) curdlan (Wako Pure Chemicals Industries, Japan) suspension prepared in distilled water on the glass coverslip, followed by 20-min thermal gelation in a waterbath at 80 °C and 90 °C. After heating, the samples were air-dried at room temperature. The thickness of the dried curdlan films was measured using electronic micrometer with accuracy 0.001 mm (Schut Geometrical Metrology, Groningen, The Netherlands). Film thickness was equal to 82 µm ± 9.6 µm.

### 4.2. Spectroscopy

A FT-IR Nicolet 8700 Continuum (Thermo Scientific, Waltham, MA, USA) spectrometer in the attenuated total reflection (ATR) mode with the GladiATR diamond crystal (PIKE Technologies) was used to record the infrared absorption spectra. To collect the IR spectra, Omnic™ 8 software from Thermo Fisher Scientific (Madison, WI, USA) was utilized. All ATR-FTIR data were obtained at room temperature in the spectral region between 4000 and 750 cm^−1^. Each spectrum was shown as an average of 120 scans with spectral resolution of 4 cm^−1^. The instrument was constantly purged with N_2_ for 40 min before and during measurements. The results are presented as an average of 3 spectra for each sample after baseline correction. In order to assess the changes in the structure of tested samples, second order derivative spectra were determined after smoothening, applying the Savitzky-Golay algorithm with nine points (Grams/AI software from ThermoGalactic Industries, Keene, NH, USA).

### 4.3. Raman Spectroscopy

The Raman measurements were carried out using a DXR confocal Raman Microscope equipped with the Omnic™ 8 software from Thermo Fisher Scientific (Madison, WI, USA) and an X-Y-Z motorized sample stage. The excitation laser wavelength was 780 nm. A Peltier-cooled CCD detector registered dispersed light was used. The experiments were performed with the use of a long working distance ×10 objective with 0.5 min photobleach time. All spectra were obtained at room temperature in the 200–2000 cm^−1^ spectral range with an exposure time of 10 s with 10 mW laser power, and 10 exposures per point with the use of an operating spectral resolution of 4 cm^−1^ of Raman shift. To determine the structural changes, second order derivative spectra were calculated using the Savitzky-Golay algorithm with thirteen points.

### 4.4. X-ray Photoelectron Spectroscopy (XPS)

X-ray photoelectron measurements (XPS) were performed using a Microlab 350 (Thermo Electron) spectrometer with non-monochromatic Al K_α_ radiation (hν = 1486.6 eV) as an X-ray source operating at 2 × 5 mm spot size, 300 W and 15 kV. The high-resolution (HR) XPS spectra were collected through hemispherical analyzer at the pass energy of 40 and the energy step size of 0.1 eV. The Avantage software (version 4.88) was used to evaluate the XPS data. Deconvolution of all HR XPS spectra were performed using a Smart-type background and a Gaussian peak shape with 35% Lorentzian character. The measured binding energies were corrected in relation to the energy of carbon peak (C1s) at 284.6 eV [27].

### 4.5. Contact Angle Measurements

The wettability and surface free energy of the matrices were assessed by the static contact angle method using DSA 30 Kruss goniometer (Kruss GmbH, Hamburg, Germany). Ultra-pure water and diiodomethane (DM, Sigma Aldrich-Chemicals) were used in the experiment as polar and non-polar liquid, respectively. The surface energies and their components (polar and disperse) were determined based on an OWRK method. For each type of matrix, three distinctive samples (*n* = 3) were prepared and each polysaccharide film was measured at least 8 times.

### 4.6. Surface Topography Evaluation

#### 4.6.1. Atomic Force Microscopy

The surface topography of the samples was imaged at a high resolution by means of atomic force microscopy (AFM, system Dimension Icon from Bruker). All images were collected in tapping mode using standard AFM probes (Bruker). For each sample, the microscopic investigations were repeated for different surface areas to gather statistical information. The presented images are representative for each sample.

#### 4.6.2. Scanning Electron Microscopy

Surface morphology of the matrices was also visualized by SEM. For this purpose, dried matrices were sputtered under a high vacuum with a 10 nm gold layer and subjected to observation in a high vacuum environment at an accelerating voltage of 5 kV using SEM (JEOL JCM-6000Plus).

## 5. Conclusions

The analysis of the molecular structure of 1,3-β-d-glucan needs the application of various microscopic and spectroscopic methods to estimate the impact of the gelation temperature. Due to some limitation of these methods applied separately, cooperative use of diverse approaches (ATR FT-IR and Raman spectroscopies, AFM, XPS, SEM and contact angle measurements) can lead to more thorough structural information of curdlan subjected to different conditions. Conducted studies showed that curdlan gelled at 80 °C possesses different characteristics than the sample manufactured at 90 °C. This gelation temperature causes visibly distinct molecular arrangement and surface structure and topography in curdlan polymers.

## Figures and Tables

**Table 1:** The most important bands obtained in the FT-IR spectra of 1,3-β-d-glucan [29,30].

Wavenumber/cm^−1^	Assignment
3300	-CONH-
2917	CH_3_, CH_2_
2884	C-H
1640	C=O
1576	COO^−^, CN
1463	CH_2_
1419	CH_2_, CH_3_
1367	CH, CH_3_
1312	CH_2_
1285	C-O (trans conformation)
1235	C-OH
1203	C-O, C-O-C
1157	C-O-C (ring)
1107	C-O
1067	C-O
1028	C-O
991	C-O, C-C
926	C-H
887	β-linked glycosidic bonds

**Table 2:** The most important bands obtained in the Raman spectra of 1,3-β-d-glucan [6,36].

Raman Shift/cm^−1^	Assignment
222	C=C (bending)
289	C-C-C (def.)
324	C-C-C-C (out of plane bending)
339	C-CH_3_ (def.)
358	C-C-C (def.)
395	C-C(=O)C (def.)
425	HCC (out of plane bending)
467	C-C=O (in plane bending)
527	C-N=C (def.)
570	C-C-C (def.)
607	CH (out of plane bending)
812	CH (out-of-plane bending)
891	HCC, HCO, CH (def. out of plane, β-glucoside bond)
938	CH (def. out of plane)
956	CH (rings) (stretch.)
998	CC, COC (stretch.)
1043	CC, COH, CH (def.)
1064	CO (stretch. sym.)
1092	CC, CO (stretch.)
1116	COC (stretch.)
1148	COC (glycosidic bonds)
1201	CCH (def.)
1227	CH rings (stretch.)
1254	CCH (def.)
1315	CH, OH (def. in plane)
1367	CH, COH (def.)
1411	CO (stretch.)
1423	O-CH_3_, CH_2_ (def.)
1461	O-CH_3_, HCH, HOC, CH (def. asym.), CH_2_ (in plane bending)

**Table 3:** C1s_,_ O1s and N1s binding energies evaluated from the corrected XPS spectra deconvolution of glucan samples.

	Binding Energy/eV High Resolution Spectra					Chemical Composition
GLU 80 °C	C1s	O1s	N1s	C:O At. % ratio (C:N)	species	O—32.6 N—2.7 Si—1.1 C—63.5 S—0.2
	284.6 286.3 287.8 289.0 282.4	533.0 531.3 535.4	400.1 401.8 398.1	1.95 (23.51)	C-C/C-H C-OH, C-N O-C-O O=C-OH Si-C-O H_2_O C-NH_x_ N_3_^−^	
GLU 90 °C	C1s	O1s	N1s	C:O At. % ratio (C:N)	species	O—30.9 N—2.8 Si—0.3 C—65.3 S—0.8
	284.6 286.2 287.8 289.1 282.7	532.8 531.3 535.8	399.9 402.0 398.1	2.11 (23.55)	C-C/C-H C-OH, C-N O-C-O O=C-OH Si-C-O H_2_O C-NH_x_ N_3_^−^	

**Table 4:** Surface free energy and its dispersive and polar components calculated for glucan matrices gelled at different temperatures.

Gelation Temperature	Surface Free Energy [mN/m] ± SD	Dispersive Part [mN/m] ± SD	Polar Part [mN/m] ± SD
Glu 80 °C	37.72 ± 1.67	34.87 ± 0.95	2.85 ± 0.72
Glu 90 °C	37.59 ± 1.64	35.01 ± 1.00	2.58 ± 0.64

## Abbreviations

ATR FT-IR	Attenuated Total Reflection Fourier Transform Infrared spectroscopy
XPS	X-ray photoelectron spectroscopy
AFM	Atomic Force Microscopy
SEM	Scanning Electron Microscopy
BSA	Bovine Serum Albumin
ATRA	All-trans Retinoic Acid
HepG2	Hepatoma cell line
